# Understanding of risk factors for the human papillomavirus (HPV) infection based on gender and race

**DOI:** 10.1038/s41598-018-36638-z

**Published:** 2019-01-22

**Authors:** Nosayaba Osazuwa-Peters, Eric Adjei Boakye, Rebecca L. Rohde, Rajan N. Ganesh, Ammar S. Moiyadi, Adnan S. Hussaini, Mark A. Varvares

**Affiliations:** 1 Saint Louis University Cancer Center, 3655 Vista Avenue, 3rd Floor West Pavilion, St. Louis, MO 63110 USA; 2 Saint Louis University School of Medicine, Department of Otolaryngology-Head and Neck Surgery, St. Louis, MO 63110 USA; 3 Saint Louis University College of Public Health and Social Justice, Department of Epidemiology, St. Louis, MO, St. Louis, MO 63104 USA; 4 Southern Illinois University School of Medicine, Springfield, IL 62794 USA; 5 Saint Louis University School of Medicine, St. Louis, MO 63104 USA; 6 Georgetown University Medical Center, Department of Otolaryngology-Head and Neck Surgery, Washington, DC 20007 USA; 7 Harvard Medical School, The Massachusetts Eye and Ear Infirmary, Department of Otolaryngology, Boston, MA 02114 USA

## Abstract

This study assessed if race and gender predict known sexual risk factors associated with HPV. Data (*n* = 301) were from a cross-sectional study conducted at a drag racing event on September 12–13, 2015 in Madison, Illinois. Both multivariable logistic and linear regression models estimated the association between race, gender, and sexual risk factors. About 63% of participants were males, and 65% identified as Blacks. Compared to females, males were more likely to have a higher number of oral sexual partners (OR = 2.10; 95% CI: 1.23, 3.57). Males were also more likely to have earlier oral sexual (b = −2.10; 95% CI: −3.60, −0.60) and vaginal sexual (b = −1.10; 95% CI: −1.69, −0.31) debuts compared to females. Blacks were more likely to have higher number of vaginal sexual partners (OR = 3.38; 95% CI: 1.81, 6.31) and earlier vaginal sex (b = −1.09; 95% CI: −1.78, −0.41) but less likely to have earlier oral sexual debuts compared with Whites (b = 2.67; 95% CI: 1.21, −4.13). Because HPV is associated with several cancers, our findings provide impetus for the development of targeted educational interventions aimed at improving the knowledge of these sexual risk factors, especially among men and across race groups.

## Introduction

Sexually transmitted infections (STIs) remain a source of significant morbidity and mortality in the United States and are strongly associated with racial and gender disparities^1^. Notably, the human papillomavirus (HPV), with its more than 150 documented strains, is the most prevalent STI in the United States, and virtually all sexually active adults will acquire HPV infection in their lifetimes^2^. Although most of these strains remain asymptomatic, a few oncogenic types, particularly HPV-16 and HPV-18, are associated with an increased risk of several anogenital cancers, including cervical, vaginal, vulvar, penile, anal, as well as oropharyngeal cancers^3^. Each year, over 43,000 newly diagnosed cancers are thought to be associated with HPV, and nearly half of these new cancer cases are HPV-positive oropharyngeal cancer^4^. HPV-positive oropharyngeal cancer has emerged as a leading HPV-associated cancer in the United States^4,5^, and its incidence has increased by 225% in the last three decades^6^.

High-risk sexual behavior is the main risk factor associated with the acquisition and persistence of HPV infection and development of HPV-associated cancers^7^. These sexual risk behaviors include age of first vaginal sex, age of first oral sex, and number of oral and vaginal sexual partners^7^. Previous studies have shown that these HPV-associated sexual risk factors vary by socioeconomic status, age, race, and education level^8^. However, there is a dearth of information about how individuals engage in these sexual behaviors in the context of HPV, based on their race and gender^9^. Efforts to investigate predictors of HPV infection in populations at high risk for HPV infection are critical to addressing healthcare disparities, especially understanding the differences in sexual behavior between males and females and between individuals from different racial backgrounds. This is important nationally as STIs continue to contribute significantly to the healthcare expenditure of the United States and remain a significant cause of mortality and morbidity^10^. It is even more important in the St. Louis region, which has consistently reported higher STI rates than more than half of major metropolitan areas in the United States reporting to the CDC^11^.

The goal of this study was to assess racial and gender differences in the patterns of high-risk sexual behaviors among a predominantly Black population surveyed at an event hosted by the United Black Drag Racers Association (UBDRA). This event draws participants from communities throughout the St. Louis metropolitan area and the Midwest region. Study aims were to assess number of oral sexual partners, number of vaginal sexual partners, age of first oral sex, and age of first vaginal sex, in association with HPV infection in this population.

## Results

Characteristics of the study participants are summarized in Table 1. A total of 301 individuals with a mean age of 48.9 ± 13.3 years completed the questionnaire. Of these, 63% were males, and 65% identified as Black. Approximately 45% of participants reported they have had five or more oral sexual partners and 77% reported they have had five or more vaginal sexual partners. Mean age for first oral sex was 19.6 ± 5.3 years and 16.6 ± 2.8 years for first vaginal sex. Compared with females, males had a higher number of oral (*p* = 0.0021) and vaginal (*p* = 0.0108) sexual partners, initiated both oral sex (*p* = 0.0181) and vaginal sex (*p* = 0.0002) at an earlier age, and were less educated (*p* = 0.0079). There was no statistically significant difference between males and females in terms of race, marital status, income, drinking and cigarette smoking status.

**Table 1:** Characteristics of survey participants, overall and stratified by sex, n = 301^$^.

Variables Mean ± SD or n (%)	Total	Gender		p-value
		Males (n = 190)	Females (n = 111)	
Age (in years)	48.9 ± 13.3	50.5 ± 13.8	46.1 ± 11.9	**0**.**005**
**Race**				
Whites	100 (33.2)	59 (31.0)	41 (36.9)	0.3000
Blacks	194 (64.5)	125 (65.8)	69 (62.2)	
Other	7 (2.3)	6 (3.2)	1 (0.9)	
**Number of oral sexual partners**				**0**.**0021**
High	135 (44.9)	98 (51.6)	37 (33.3)	
Low	166 (55.1)	92 (48.4)	74 (66.7)	
**Number of vaginal sexual partners**				**0**.**0108**
High	233 (77.4)	156 (82.1)	77 (69.4)	
Low	68 (22.6)	34 (17.9)	34 (30.6)	
Age at first oral sex (in years)	19.6 ± 5.3	19.0 ± 5.4	20.7 ± 5.0	**0**.**0181**
Age at first vaginal sex (in years)	16.6 ± 2.8	16.2 ± 3.0	17.3 ± 2.3	**0**.**0002**
**Marital status**				0.6095
Married	167 (55.5)	104 (54.7)	63 (56.8)	
Single but Dating	77 (25.6)	52 (27.4)	25 (22.5)	
Single and not Dating	57 (18.9)	34 (17.9)	23 (20.7)	
**Education**				**0**.**0079**
College graduate or higher	63 (20.9)	30 (15.8)	33 (29.7)	
Some College	81 (26.9)	49 (25.8)	32 (28.8)	
Associate Degree	54 (17.9)	35 (18.4)	19 (17.1)	
High school diploma or less	103 (34.2)	76 (40.0)	27 (24.4)	
**Income**				0.0675
≥100,000	46 (15.3)	36 (19.0)	10 (9.0)	
75,000–99,999	48 (16.0)	27 (14.2)	21 (18.9)	
50,000–74,999	79 (26.3)	50 (26.3)	29 (26.1)	
25,000–49,999	74 (24.6)	49 (25.8)	25 (22.5)	
<25,000	54 (17.9)	28 (14.7)	26 (23.4)	
**Drinking status**				0.0582
Never drinker	46 (15.3)	22 (11.6)	24 (21.6)	
Former drinker	36 (12.0)	25 (13.2)	11 (9.9)	
Current drinker	219 (72.7)	143 (75.2)	76 (68.5)	
**Cigarette smoking status (excluding e-cigarettes)**				0.1287
Never smoker	182 (60.5)	109 (57.4)	73 (65.8)	
Former smoker	51 (16.9)	31 (16.3)	20 (18.0)	
Current smoker	68 (22.6)	50 (26.3)	18 (16.2)	

^$^Number of oral and vaginal sexual partners was classified as high meaning five or more and low as less than five, according to measures described by the National Health Statistics Report.

### Number of oral and vaginal sexual partners

Multivariable logistic regression, summarized in Table 2, revealed that gender was a significant predictor of number of oral sexual partners, but race was not. Males compared with females were more likely to have a high number of oral sexual partners (aOR = 2.10; 95% CI: 1.23, 3.57). There was no statistically significant difference between the race groups and number of oral sexual partners. Race was a significant predictor of number of vaginal sexual partners, but gender was not. Blacks were more likely to have a higher number of vaginal sexual partners (OR = 3.38; 95% CI: 1.81, 6.31) compared with Whites. There was no statistically significant difference between males and females with respect to number of vaginal sexual partners.

**Table 2:** Multivariable logistic regression models for number of oral and vaginal sexual partners in relation to sex and race and covariates, (n = 301).

	Number of oral sexual partners		Number of vaginal sexual partners	
	^a^OR	95% CI	^a^OR	95% CI
Age (in years)	0.98	0.94–1.00	1.01	0.99–1.04
Gender				
Females	Ref	Ref	Ref	Ref
Males	2.1	1.23–3.57	1.54	0.84–2.80
Race				
Whites	Ref	Ref	Ref	Ref
Blacks	0.79	0.47–1.35	3.38	1.81–6.31
Other	5.03	0.53–47.9	3.2	0.34–29.9
Marital status				
Married	Ref	Ref	Ref	Ref
Single but Dating	1.81	0.96–3.42	1.82	0.81–4.12
Single and not Dating	1.17	0.57–2.40	0.95	0.43–2.13
Income				
≥100,000	Ref	Ref	Ref	Ref
75,000–99,999	1.04	0.44–2.48	0.41	0.12–1.37
50,000–74,999	0.44	0.20–0.99	0.23	0.08–0.71
25,000–49,999	0.78	0.33–1.85	0.39	0.12–1.32
<25,000	0.5	0.19–1.34	0.19	0.05–0.69
Drinking status				
Never drinker	Ref	Ref	Ref	Ref
Former drinker	1.43	0.53–3.88	1.28	0.42–3.86
Current drinker	1.64	0.78–3.42	1.62	0.75–3.50
Cigarette smoking status				
Never smoker	Ref	Ref	Ref	Ref
Former smoker	1.05	0.52–2.14	1.51	0.66–3.42
Current smoker	1.06	0.56–2.00	2.63	1.09–6.32

^a^OR = adjusted odds ratgio, CI = confidence interval.

### Age at first oral and vaginal sex

In multivariable linear regression analysis, summarized in Table 3, gender and race were significant predictors of age at first oral sex. Males (b = −2.10; 95% CI: −3.60, −0.60) were more likely to have earlier oral sexual debut as compared with women. Whites were more likely to have earlier oral sexual debut as compared to Blacks (b = 2.67; 95% CI: 1.21, 4.13). In regard to age at first vaginal sex, males (b = −0.10; 95% CI: −1.69, −0.31) were more likely to have earlier vaginal sexual debut compared with females as were Blacks (b = −1.09; 95% CI: −1.78, −0.41) compared with Whites. Participants who were current cigarette smokers (b = −1.19; 95% CI: −2.02, −0.36) were more likely to have earlier vaginal sexual debut compared with never smokers.

**Table 3:** Multivariable linear regression models for age at first oral and vaginal sex in relation to sex and race and covariates, (n = 301)^$^.

	Age at first oral sex		Age at first vaginal sex	
	Adjusted b	95% CI	Adjusted b	95% CI
Gender				
Females	Ref	Ref	Ref	Ref
Males	−2.1	−3.60, −0.60	−0.1	−1.69, −0.31
Race				
Whites	Ref	Ref	Ref	Ref
Blacks	2.67	1.21, 4.13	−1.09	−1.78, −0.41
Other	0.1	−4.28, 4.48	−1.23	−3.32, 0.86

CI = confidence interval.

^$^Model is adjusted for age, gender, race, marital status, income, dinking status and cigarette smoking status.

## Discussion

This study aimed at understanding the racial and gender differences in sexual risk-taking behaviors among individuals in a primarily Black community, and our findings indicate that there are considerable racial and gender differences in high-risk sexual behaviors that are associated with HPV infection. While male participants were more likely to initiate both vaginal and oral sexual intercourse earlier than women, Whites were more likely than Blacks in general to initiate oral sex. These racial and gender patterns shown in our study align with current trends in the incidence of HPV-associated cancers of the oropharynx and have important implications in their prevention. Recent data suggest that the rate of HPV-associated oropharyngeal cancer ranged from 3.5 to 4.8 times higher for men than for women, but significantly lower among Blacks than Whites^11^. The data derived from this study has importance for educating members of the community about cancer prevention through HPV vaccination.

Findings that demonstrated a significant difference in age of oral and vaginal sexual debut based on gender are consistent with previous studies that have shown that males, more than females, report earlier initiation of sexual activity^12^. On average, males in the United States report sexual debut at 16.8 years, females at 17.2 years^12^. Of note, much of the published data on sexual debut limits the definition of sexual intercourse to vaginal penetration, forgoing an important, yet commonly overlooked, practice that is oral sexual intercourse^12^. This study extends the scope of the literature by classifying sexual debut as age at either first vaginal or first oral intercourse. It is important to distinguish the role of gender differences in not only vaginal but also in oral sexual practices, because studies have reported that men are at a three-fold higher risk of developing HPV-associated head and neck cancers compared to females^13^. Oral HPV is a major etiological factor in the development of head and neck squamous cell cancers, particularly oropharyngeal cancer^14^, and linked to oral sexual practices^2,3,14^. Because males are more likely to initiate oral sex and they also have a higher burden of HPV-associated oropharyngeal cancer, it is important that males in the community be educated about their oral sex lifestyles and their higher risk of developing HPV-associated oropharyngeal cancer.

Oral sex behavior is well-established as a central driver of the increasing incidence of HPV-associated oropharyngeal cancer, which has now replaced cervical cancer as the leading HPV-associated cancer^3,15^. Because individuals who initiate oral and vaginal sex at an early age, and/or have a greater number of oral sexual partners have higher risks of developing oropharyngeal cancer^2,3,13^, our findings have important implications for the community, especially among males. While cervical cancer continues to contribute to the overall cancer burden of the United States, the incidence and mortality rates have decreased tremendously in the last five decades^14^. Cervical cancer is also limited to females, whereas HPV-associated oropharyngeal cancers not only affect both genders, they, in fact, disproportionately affect males^16^. Cervical cancer prevention and control has also benefitted strongly from the availability of an early detection tool, the Papanicolaou (Pap) test^14^. Its introduction in the 1940’s allowed for earlier detection of cervical precancerous lesions and has greatly contributed to the dramatic decrease in both the incidence and mortality rates related to cervical cancer^14^. However, similar screening mechanisms for precancerous lesions of the oropharynx, as part of a periodic health examination, have not been established, although there have been calls for such a mechanism to be established^17^. For this reason, greater focus on oropharyngeal cancer prevention by means of increasing awareness about risky sexual activities is warranted as the next best prevention strategy.

Currently, the position of the United States Preventive Services Task Force (USPSTF) is that there is inadequate evidence to establish mortality benefits for mass oral cancer screening for asymptomatic individuals^16^. This lack of clear evidence of benefits of mass oral cancer screening places greater value on HPV vaccination as an important means of preventing HPV-associated oropharyngeal cancer. All three HPV vaccines (bivalent, quadrivalent, nonavalent) presently in use are highly efficacious in providing long-term immunity against various strains of HPV, including the oncogenic types 16 and 18 which represent 90–95% of HPV-associated oropharyngeal cancers^18^. Previous HPV vaccine trials have demonstrated nearly 100% response by oral HPV strains to existing HPV vaccines^19^. However, vaccination coverage among American youth remains low. Currently, the percentage of female adolescents receiving all three doses of HPV vaccine by age 13–15 years was documented at 37.1%, whereas only 27.1% of males in the same age category completed the full three-dose series^20^. Although the complete dose of HPV vaccine has since been updated from three doses to two if taken before 15 years of age^21^, the persistently low HPV vaccine uptake render the *Healthy People 2020* objective of achieving 80% uptake highly unlikely^22^. Not only would increasing vaccination coverage of males protect against transmission of oral HPV infection and effectively reduce the incidence of HPV-associated oropharyngeal cancers, but it would also imply a secondary protective effect for individuals who have not been vaccinated, through a phenomenon known as herd immunity^23^. Therefore, the results of our study identify an objective need for male-targeted HPV vaccination campaigns, as well as guided sexual health programs that are more conscious of the role that gender plays in sexual debut at a younger age.

Besides males having lower HPV vaccination uptake, other studies have shown that males have poorer knowledge of the association between HPV and oropharyngeal cancer, despite the fact that males are indeed at significantly higher risk for developing head and neck cancer than females^6^. Thus, while the USPSTF position on mass oral cancer screening is clear, this lack of knowledge about risk factors for head and neck cancer, such as HPV, has been cited as a rationale for providing educational opportunities for lay members of the public through oral cancer screening.

Differences in sexual practices vary by race and ethnicity and may shift over time as a reflection of societal influences on sexual behavior^24^. In the present study, our results found that Blacks, when compared to Whites, were more likely to have a higher number of vaginal sexual partners rather than oral sexual partners. These findings are consistent with previous studies which found that White youths tend to engage in oral sex more often than Black youths and that Black girls and boys tend to initiate oral sex at a later age than Whites, after having initiated vaginal sex at an earlier age^25^. There may be cultural or societal norms driving this racial pattern in initiating oral and vaginal sex shown in our study. A previous nationally representative study showed that Whites were more likely than Blacks to engage in oral sex and perceive it as nonsexual, less intimate, or serious^26^. Additionally, authors found that only 55% of Black males and 25% of Black females reported having oral sex as appealing, compared to 66% of White males and 55% of White females^26^. Because the increasing incidence of HPV-associated oropharyngeal cancer has been driven largely by White males^6^, this finding that Whites initiate oral sex earlier highlights the need to continue to increase knowledge about the association between oral sex, oral HPV, and HPV-associated oropharyngeal cancer.

It is well known that other high-risk behaviors impact an individuals’ risk for acquiring HPV infection, including cigarette smoking^27^. Results of this study parallel those of previous studies, having revealed that current smokers were more likely to engage in earlier sexual behavior^28^. These findings are perhaps suggestive that smoking behavior is a marker for other high-risk behaviors, including sexual activity. High-risk sexual behaviors, if established in adolescence, are likely to extend into adulthood and have serious implications for the risk of acquiring HPV infection. Given this established association between early sexual debut and substance abuse behaviors, interventions that address both risk domains are more likely to result in greater influence on positive behavior change than would focusing on either high-risk behavior.

Finally, this study adds value to the current literature through its innovative methodology. Survey of community members in a non-traditional setting proved useful for carrying out this research effectively. For some interventions, particularly those targeting high-risk populations, selection of an appropriate setting may seem less obvious. Therefore, identification of a potential setting that was both convenient and comfortable for our study participants was a strategic action employed in the planning phase of this investigation.

Additionally, application of electronic data capture for survey distribution offered logic features not otherwise available with the use of paper surveys. These design capabilities permitted customization of the survey to each participant and prevented submission of incomplete responses. Moreover, use of this questionnaire platform was less time-consuming and less costly compared with the use of a paper-based questionnaire. Prior studies have reported that use of Web-based questionnaires maintains similar, if not greater, accuracy of reported information, as well as fewer response errors and socially desirable responses compared to paper-based surveys^29,30^. Thus, integration of electronic data capture for this study purpose offered an attractive alternative within this population.

This study has several limitations that should be noted. First, the relatively small sample size of our study may have prevented the detection of other significant differences among the participants. However, our sample size is consistent with other community-based studies at race events^31–34^. Second, the data was collected from a convenience sample within a narrow population; therefore, our results are susceptible to selection bias. The possibility of recall and social desirability bias also deserves mention, as this study asked participants to recall sexual risk behaviors from their late adolescence to young adulthood – nearly three decades prior. As such, applicability of this survey to the current population that is at sexual debut will be more beneficial from an interventional and cancer prevention standpoint. Additionally, data regarding anal sex and oral sex, stratified by sexual orientation and practice (receptive vs. insertive) was not collected; however, future studies should include this information for the purpose of designing effective interventions in this population. This type of data concerning minority sexual practices, which are highly stigmatized in this population, may best be collected in a qualitative setting using focus groups or structured interviews^35^. Importantly, specific sexual practices (i.e., inceptive or receptive sex) may have different implications for enhancing the risk of HPV acquisition within the oral cavity.

Furthermore, most participants in our study were Black males; therefore, limiting the generalizability of our findings to the US population. However, because studies on demographic factors as predictors of risk of HPV infection are limited and predominantly focus on women, information from male participants, who are more susceptible to HPV-associated oropharyngeal cancer, is invaluable in identifying gender gaps to tailor methods to improve behavioral interventions. Though the generalizability of this study is limited by characteristics of the study sample, its conclusions prompt the need for future studies to include measures of HPV vaccination status, as well as knowledge and awareness of HPV and HPV-associated oropharyngeal cancer to better identify racial-gender gaps that may exist.

## Conclusions

Our study showed that race and gender are independent predictors of HPV risk factors, and being male, and White were found to be risk factors for initiating earlier oral sex, a sexual behavior closely associated with HPV infection and HPV-associated oropharyngeal cancer. Our findings provide impetus for the development of targeted educational interventions that are sensitive to gender and racial differences in sexual risk behaviors. The need for preventive programs to improve knowledge of males about sexual risk factors and their link to cancer is paramount in the community.

## Methods

The study was approved by the Saint Louis University Institutional Review Board. All procedures performed in studies involving human participants were in accordance with the ethical standards of the institutional and/or national research committee and with the 1964 Helsinki declaration and its later amendments or comparable ethical standards. This was a cross-sectional study conducted at the 2015 United Black Drag Racers Association event on September 12 and 13, 2015, in Madison, Illinois. Established in 1994, the UBDRA has gained national recognition as one of the premier Black organizations in the world of motorsports. Study participants consisted of UBDRA drag racers, vendors, and spectators from across the Midwest region who attended the drag race event. Participants responded to a 78-item questionnaire survey that was based on previously validated surveys^36^. The questionnaire was administered using research electronic data capture (REDCap) methods and software tools hosted at Saint Louis University^35^. REDCap is a secure, web-based application designed to support data capture for research studies providing (1) an intuitive interface for validated data entry, (2) audit trails for tracking data manipulation and export procedures, (3) automated export procedures for seamless data downloads to common statistical packages, and (4) procedures for importing data from external sources^35^. A total of 15 trained volunteer survey administrators approached all participants around the racing area, including spectators in the bleachers, vendors at concession stands, and drag racers in the pit area. Potential enrollees were informed of the anonymous nature of the survey and the objective of the study, and oral informed consent was obtained from all participants. The data collected represents a convenience sample of 301 participants, 18 years or older. In exchange for participation, subjects were offered the opportunity to be entered anonymously into a raffle drawing of 15 various prizes, valued at no more than $20 each.

### Measures

Questions elicited sociodemographic factors including age, gender, race, marital status, education, income, drinking status, cigarette smoking status, as well as sexual factors including number of oral and vaginal sexual partners, age of first vaginal sex, and age at first oral sex. The independent variables assessed in this study included gender and race and were categorized as follows: race (White, Black or other [which combined low-frequency responses]), and gender (male or female).

The outcome variables assessed in this study included number of oral and vaginal sexual partners, age of first vaginal sex, and age of first oral sex. To collect this data, the following questions were asked: *1) At what age did you FIRST have vaginal sexual intercourse? (Please list age in years) 2) How many vaginal sexual partners have you had in your lifetime? 3) At what age did you FIRST have oral sexual intercourse? (Please list age in years) 4) How many oral sexual partners have you had in your lifetime?* Number of sexual partners was categorized as follows: number of oral and vaginal sexual partners was classified as *high* vs. *low* with five or more representing a ‘high’ number, according to measures described by the National Health Statistics Report.

Covariates adjusted for included: age; marital status (married, single but dating, and single and not dating); education level (college graduate or higher, some college, associate degree, and high school diploma or less); income level (≥100,000, 75,000–99,999, 50,000–74,999, 25,000–49,999, <25,000); drinking status (never drinker, former drinker, current drinker); and cigarette smoking status [excluding e-cigarettes] (never smoker, former smoker, current smoker).

### Statistical Analysis

Analyses were performed using statistical software (SAS, version 9.4; SAS Institute Inc, NC). Descriptive statistics (frequency and percentage) were used to analyze characteristics of the respondents. We compared sociodemographic characteristics and sexual behavioral factors by using χ^2^ tests for categorical variables and independent sample t tests for continuous variables. Multivariable logistic regression was used to examine the association between race and gender, and number of oral and vaginal sexual partners. Multivariable linear regression analysis assessed the association between race and gender, and age of first vaginal sex, and age of first oral sex. Analyses were 2-tailed, and statistical significance was set at *p* < 0.05.

## Data Availability

Public access to the data is restricted; however, data may be available on request with permission from the United Black Drag Racers Association.
